# Pathogenic Roles of the Carotid Body Inflammation in Sleep Apnea

**DOI:** 10.1155/2014/354279

**Published:** 2014-09-07

**Authors:** Man Lung Fung

**Affiliations:** Department of Physiology, The University of Hong Kong, 21 Sassoon Road, Pokfulam, Hong Kong

## Abstract

Breathing difficulties in sleep are a hallmark of sleep-disordered breathing commonly observed in patients with sleep disorders. The pathophysiology of sleep apnea is in part due to an augmented activity of the carotid body chemoreflex. Arterial chemoreceptors in the carotid body are sensitive to inflammatory cytokines and immunogenic molecules in the circulation, because cytokine receptors are expressed in the carotid body in experimental animals and human. Intriguingly, proinflammatory cytokines are also locally produced and released in the carotid body. Also, there are significant increases in the expression of proinflammatory cytokines, cytokine receptors, and inflammatory mediators in the carotid body under hypoxic conditions, suggesting an inflammatory response of the carotid body. These upregulated cytokine signaling pathways could enhance the carotid chemoreceptor activity, leading to an overactivity of the chemoreflex adversely effecting breathing instability and autonomic imbalance. This review aims to summarize findings of the literature relevant to inflammation in the carotid body, with highlights on the pathophysiological impact in sleep apnea. It is concluded that local inflammation in the carotid body plays a pathogenic role in sleep apnea, which could potentially be a therapeutic target for the treatment of the pathophysiological consequence of sleep apnea.

## 1. Introduction

Sleep apnea is the most prevalent form of sleep-disordered breathing, which is characterized by recurrent breathing pauses lasting over 10 seconds due to a repeated narrowing or closure of the upper airway and/or a periodic halt of the central drive for respiratory activities during sleep. Sleep apnea syndrome is closely associated with episodes of desaturated arterial blood oxygen, sleep fragmentation, and arousals in the patient. As a consequence of sleep apnea, it causes excessive daytime sleepiness and increases the risk of neurocognitive dysfunction and cardiovascular morbidities in young and adult patients [1, 2]. The pathophysiology and consequences of sleep apnea have been extensively studied over decades. A growing amount of evidence suggests that the carotid chemoreflex elicited by arterial chemoreceptors in the carotid body in response to episodes of hypoxia (intermittent hypoxia) in sleep apnea plays a role in the pathophysiological cascade [3]. Specifically, there are significant increases in the chemosensitivity and the activity of the carotid chemoreceptor induced by hypoxia in experimental animals and in patients with sleep apnea [4, 5]. Thus, mechanisms underlying the augmented activity of the carotid chemoreceptor are currently a focus of investigation.

Arterial chemoreceptors in the carotid body play an important physiological role in the chemotransduction of chemical changes in the arterial blood, which is essential to elicit the chemoreflex for rapid adjustment of respiratory and cardiovascular activities to maintain blood gases and pH homeostasis. Thus a lowered arterial oxygen or pH level causes an increase in the chemoreceptor activity of the carotid body [6]. The increased afferent activity via the carotid sinus nerve projects to the nucleus tractus solitarius in the medulla, through which the primary relaying neurons activate the central pathway of the chemoreflex. This in turn increases the central drive for ventilation and also alters cardiac and autonomic activities for the compensatory changes adjusting to metabolic needs [7].

The carotid body is bilaterally located at the bifurcation of the carotid artery, which is structurally analogous to vagal paraganglia. The major cell type in the carotid body is type-I glomus cells, which are chemosensitive and responsive to chemical changes in the arterial blood. These cells form lobular clusters and are apposed to afferent nerve endings, which are essential for the sensory chemotransduction [6, 8]. These glomic clusters are encapsulated by type-II sustentacular cells, which are glial like and are recently known to play a role in the paracrine signaling in the chemotransduction and other housekeeping functions [9]. In responding to hypoxia, chemosensitive type-I glomus cells are depolarized with an inhibition of potassium currents causing an elevated level of intracellular calcium, which triggers the vesicular release of neurotransmitters acetylcholine and ATP as well as neuromodulators including catecholamines and neuropeptides from the chemosensitive cells [10]. This elevates the excitability of the nerve endings which increases the activity of the carotid sinus nerve of the carotid body for eliciting the chemoreflex.

The carotid body is highly vascularized with blood perfusion from the carotid artery far more than the local metabolism [6]. Thus, the chemosensory component of the carotid body is in a close diffusion distance responding to any changes in arterial oxygen tension or pH, circulating hormones or signaling molecules, and also substances locally produced by vascular cells and tissues acting as autocrines or paracrines. For example, signaling molecules in the arterial blood and locally produced in the carotid body including vasoactive peptides endothelin-1, angiotensin II, and also proinflammatory cytokines have been shown to modulate the excitability of the carotid chemoreceptor [11, 12]. Also, the cytokine receptors are functionally expressed in the carotid body [13–15]. These findings provide initial evidence for a role of cytokine signaling in the carotid body, which raises a number of questions on the following: (i) the physiological significance of the expression of cytokines and cytokine receptors in the carotid body, (ii) the regulation of the cytokine signaling pathway in the carotid body under physiological or pathophysiological conditions, and (iii) alterations of the carotid chemoreceptor activity by cytokines and the pathophysiological significance of carotid body inflammation in disease conditions.

## 2. The Carotid Body Is a Peripheral Sensor of Inflammatory Cytokines

Both the central and peripheral nervous systems play roles in the communication between the brain and the immune system, which is important for the regulation of immune and brain responses to inflammation under physiological or disease conditions. The circumventricular organs are important structures where central neurons respond to cytokines in the blood, whereas the visceral activity of the vagus nerve is crucial to the afferent projection of immunogenic signals to the central [16]. In addition, recent studies suggest a role of the peripheral chemoreceptor in the carotid body in the immunosensing of inflammatory cytokines, by which the elicitation of the activity of the chemoreflex pathway could be an important part of the ventilatory response and altered cardiovascular and autonomic activities adjusting to the inflammatory status.

It is well known that the biosynthesis and release of inflammatory cytokines, including interleukin- (IL-) l*β* and IL-6 and tumor necrosis factor- (TNF-)* α* in the immune cells play important roles in the peripheral mediation of the physiological and pathophysiological changes under inflammatory and/or infective conditions. In experimental animals, it has been reported that IL-1 type-1 receptors are expressed in glomus cells of vagal paraganglia [17]. Also, the IL-1 receptors are expressed in the type-I glomus cells of the carotid body, suggesting an immunosensory function of the carotid body [13, 15, 18]. Functionally, extracellular application of IL-1*β* significantly decreases the outward potassium current in cultured glomus cells of the carotid body [19]. In addition, topical application of IL-1*β* to the carotid body increases the activity of the carotid sinus nerve in rats [19]. Also, exogenous IL-1*β* induces an elevated level of intracellular calcium response to hypoxia in the glomus cell of the carotid body [15]. Besides the expression of IL-1 receptors, components of the IL-6 receptor, namely, IL-6 receptor alpha chain and gp130, are expressed in the type-I glomus cell and possibly in type-II and vascular cells of the rat carotid body (Table 1). These receptors are functional because exogenous application of IL-6 to the glomus cells increases the intracellular calcium response to hypoxia [15]. These observations support a role of the carotid body in sensing proinflammatory cytokines via the cytokine receptors.

In addition to proinflammatory cytokines IL-1*β* and IL-6, Fernández et al. [20] reported that infusion of lipopolysaccharide (LPS) increases respiratory rate in the cat, which is abolished by sectioning the carotid and aortic nerves. The carotid chemoreceptor activity is also increased by the intravenous LPS administration [20]. The carotid chemoreceptor response to LPS could be mediated by toll-like receptor 4, which is the LPS canonical receptor, expressed in the carotid body. Also, LPS induces the release of TNF-*α* from immune cells [21, 22]. It raises the possibility that the effect of LPS may be mediated by TNF-*α*. Indeed, the TNF receptors, namely, TNF-r1 and TNF-r2, are expressed, respectively, in the type-I glomus cell and endothelial cell of the cat carotid body [20]. In the rat, the TNF receptors are also expressed in the type-I glomus cell and possibly type-II and vascular cells of the carotid body (Table 1). Exogenous application of TNF-*α* to the rat glomus cell increases the intracellular calcium response to hypoxia [15]. However, TNF-*α* attenuates the carotid chemosensory response to hypoxia in the cat [20]. Nevertheless, these findings support a role of the glomus cells in sensing and transmitting immune signals by responding to the proinflammatory cytokines via their corresponding receptors.

In human, inflammation of the carotid body has been reported to be related to aging or autoimmunity disease. The histological feature of carotid glomitis is associated with follicles of lymphocytes and also infiltration of immune cells with morphological changes in the type-I and type-II cells in the carotid body [23]. More recently, cytokine receptors for IL-1*β*, IL-6, IL-10, and TNF-*α* as well as toll-like receptors are also found to be expressed in the human carotid body [24, 25]. These findings are consistent with the findings in experimental animals, suggesting an immunosensing function of the carotid body in its response to proinflammatory cytokines and also other immunogenic molecules via the corresponding receptors expressed in chemosensitive type-I glomus cells and also other cell types in the organ.

## 3. Regulation of the Expression of Proinflammatory Cytokines and Cytokine Receptors in the Carotid Body

The carotid body and its chemoafferent activity could play a role in the communication between the immune system and the brain, in particular, in the inflammatory and infective status [26]. It raises the possibility that the cytokine signaling pathway in the carotid body may also be regulated by the inflammatory cytokines and immunogenic molecules in the circulation. Indeed, it has been shown that intraperitoneal injection of IL-1*β* increases the expression of IL-1 type-I receptors in the carotid body of the rat [27]. In addition, infusion of LPS increases the expression of TNF-r2 receptor in the rat carotid body [28]. These observations suggest that the cytokine receptors in the carotid body are regulated by inflammatory cytokines, which could modulate the chemosensory activity and its response to proinflammatory cytokines or hypoxia under inflammatory or disease conditions.

Besides the circulating source of cytokines, a growing amount of evidence supports that there is a local expression of proinflammatory cytokines in the carotid body under physiological or disease conditions. Fernández et al. [20] reported the expression of TNF-*α* is particularly significant following intravenous injection of LPS to induce endotoxemia in the cat and the expression is colocalized to the type-I and endothelial cells in the carotid body. This study provides evidence suggesting that the TNF signaling pathway in the carotid body, via activation of the chemoreflex, could be functionally important in the ventilatory and cardiovascular response to sepsis [26]. In the rat, the expression of TNF*α* has also been shown in the carotid body under physiological and hypoxic conditions (Table 1). In addition, proinflammatory cytokines IL-1*β* and IL-6 are expressed in type-I glomus cells (Table 1) and also in type-II cells of the carotid body [29, 30]. Furthermore, cytokines IL-1*β*, IL-4, IL-6, IL-8, and IL-10 are reported to be released from carotid bodies in patients with head and neck neoplasms [24]. Importantly, the release of the cytokines is increased following a prolonged period of hypoxia for an hour [24]. These findings suggest that cytokines locally produced in the carotid body are an active component of the cytokine signaling pathway which modulates the activity of chemoreceptors under physiological or hypoxic conditions.

## 4. Sustained Hypoxia Induces Local Inflammation in the Carotid Body

Inflammation plays an important role in physiological processes, for instance, wound healing, and also a common clinical condition manifested in a chronic manner in diseases. In addition to infection and inflammation, evidence suggests that the cytokine signaling pathway in the carotid body is regulated by hypoxic conditions. In the circulation, plasma levels of cytokines are elevated in altitude natives and subjects sojourning at high altitude under a sustained hypoxic condition [31–33]. Also, chronic inflammation is one of the important clinical manifestations in patients with diseases associated with chronic hypoxemia, including chronic obstructive pulmonary disease [34, 35]. In fact, hypoxia and inflammation are interrelated and the inflammatory response to hypoxia can be adaptive or pathogenic in nature [36]. It is well known that inflammatory cytokines induced the expression of nuclear factor kappa B- (NF*κ*B-) dependent genes encoding inflammatory mediators including cyclooxygenases and inducible nitric oxide synthase (iNOS), which could in turn modulate the expression of cytokines and chemokines. In this regard, the expression of cytokines and cytokine receptors in the carotid body may play roles in the physiological acclimatization to altitude, particularly in the modulation of carotid chemoreceptor activity, and also in the pathogenic cascade in disease conditions associated with chronic hypoxemia and inflammation.

We and others have shown that hypoxia induces increased expressions of proinflammatory cytokines IL-1*β*, IL-6, and TNF-*α* and the corresponding IL-1*β* receptor (IL-1r1), IL-6 receptor (gp130), and TNF receptor (TNF-r1) in the carotid body of rats exposed to sustained hypoxia for days up to 4 weeks [15, 29]. The upregulated expression of proinflammatory cytokines and cytokine receptors is localized to the type-I glomus cell and is also expressed in the type-II cell and immune cells in the carotid body in sustained hypoxia [15, 29]. Importantly studies show that exogenous cytokines induce an enhanced intracellular calcium response to hypoxia in the glomus cell of rats exposed to sustained hypoxia, suggesting that the upregulated expression of proinflammatory cytokines and cytokine receptors plays a functional role in the chemosensory function [15]. Also, the enhanced carotid chemoreceptor activity induced by sustained hypoxia is significantly blocked by a concurrent treatment of the animal with an anti-inflammatory drug ibuprofen or dexamethasone [29]. Furthermore, ibuprofen prevents the increase in the ventilatory response to hypoxia in rats exposed to sustained hypoxia [37]. These findings strongly supported the notion that the activity of chemosensitive glomus cells is enhanced by the locally produced proinflammatory cytokines, mediated by the cytokine receptors in a paracrine-autocrine signaling manner. In effect, the cytokine signaling pathway plays a more prominent role in the enhanced chemosensory activity under hypoxic conditions with an upregulation of the local expression of proinflammatory cytokines and cytokine receptors.

Evidence supports that activation of the cytokine pathway could lead to inflammation of the carotid body associated with an increased expression of inflammatory mediators and an enhanced carotid chemoreceptor activity under sustained hypoxic conditions. Indeed, the expression level of the inflammatory mediator iNOS is significantly increased in the carotid body of rats exposed to sustained hypoxia [15], which could be functionally significant in being involved in the endogenous production of NO in the carotid body under hypoxic conditions [38]. Activation of NF-*κ*B pathway may be involved in the mechanistic aspects of the cytokine-induced iNOS expression and the hypoxia-induced inflammatory response of the carotid body [15, 29]. Furthermore, sustained hypoxia induces the expression of chemokines (monocyte chemoattractant protein- (MCP-) 1, chemokine receptor- (CCR-) 2, macrophage inflammatory protein- (MIP-) 1*α*, and MIP-1*β*) and adhesive molecule (intercellular adhesion molecule- (ICAM-) 1) as well as infiltration of immune cells in the carotid body of the rat [15, 29]. Anti-inflammatory drug ibuprofen or dexamethasone attenuates the infiltration of macrophages and the expression of cytokines and chemokines in the carotid body [29]. These findings give support to the notion that the upregulated expression of the cytokines and cytokine receptors significantly contributes to the local inflammation of the carotid body in sustained hypoxia.

Besides the upregulation of cytokines and cytokine receptors, the inflammatory response of the carotid body to sustained hypoxia could be mediated by endothelin-1. Liu et al. [39] reported that pharmacological blockade of the endothelin receptors with bosentan neutralizes the elevated expression of proinflammatory cytokines, chemokines, and macrophage infiltration in the carotid body. The expression of endothelin-1 and endothelin receptors in the carotid body is significantly upregulated in the sustained hypoxia [40, 41]. Thus, as aforementioned, inflammation of the carotid body is likely to be mediated by locally regulated paracrine-autocrine signals during sustained hypoxia. In addition, it is known that plasma levels of cytokines are elevated under hypoxic conditions. The circulating level of cytokines could also be an important stimulus to induce the upregulation of the cytokine signaling pathway in the carotid body, leading to the local inflammation.

Moreover, inflammation of the carotid body is associated with an increased expression of acid-sensitive iron channels (ASIC) in the chemoafferent neurons under sustained hypoxia, hinting an altered chemoreceptor function under a hypoxia-induced inflammatory condition resembling a condition of hyperalgesia/hyperexcitability induced by chronic pain/inflammation [42, 43]. Liu and colleagues [42] reported that inflammatory cytokines significantly increase ASIC expression in cultured petrosal ganglionic neurons, which are the chemoafferent neurons of the carotid body. Also the elevated ASIC expression in the animal exposed to sustained hypoxia is blocked by concurrent treatment of a nonsteroidal anti-inflammatory drug ibuprofen. Importantly, the hypoxia-induced enhanced carotid chemoreceptor activity recorded from the carotid sinus nerve is significantly attenuated by an ASIC antagonist A-317567 and also by ibuprofen. These findings underscore an important role of ASIC in the carotid body inflammation, which leads to an enhanced carotid chemoreceptor activity under sustained hypoxic conditions. Thus the inflammatory response of the carotid body to sustained hypoxia may be an adaptive response of the carotid chemoreceptor to facilitate the ventilatory acclimatization to hypoxia at high altitude and also to adjust the ventilatory and autonomic activities in response to hypoxia under disease conditions.

In summary, evidence suggests that the increased expressions of cytokines and cytokine receptors play a role in the local inflammation of the carotid body induced by sustained hypoxia. In effect, activation of the cytokine signaling pathway increases the expression of inflammatory mediators and chemokines, which mediates the local inflammatory response of the carotid body. Thus, this upregulation could increase the locally produced cytokines in addition to the rise of circulating cytokines under hypoxic conditions, leading to activation of inflammatory signaling pathways. The chemosensitive type-I glomus cell plays an important role in this local inflammation because of the expression of the cytokines and the cytokine receptors. The elevation of cytokine levels could alter the response of the glomus cells to hypoxia, which may be one of the cellular mechanisms underlying the altered functions of the carotid body relevant to the cardiopulmonary control under sustained hypoxic conditions during physiological acclimatization to altitude and also in diseases associated with chronic hypoxemia.

## 5. Pathogenic Role of the Chemoreflex in Sleep Apnea

Recurrent apnea in patients suffering from sleep apnea leads to intermittent hypoxia, which adversely impact the neurocognitive and cardiovascular functions and increased risks for stroke and cardiovascular disease [1, 2]. The physiological compensatory response to arterial oxygen desaturation in sleep apnea is significantly mediated by the chemoreflex which triggers hyperventilation, parasympathoexcitation contributing to bradycardia, and increased sympathetic activities for the redistribution of blood flow in tissues and organ [7]. However, repeated episodes of hypoxia induce augmented activities of the carotid chemosensory activity and ventilatory hypoxic responses [44, 45]. Mounting evidence supports that the augmented activity of the carotid body induced by intermittent hypoxia plays an important role in the pathogenesis of sleep apnea [46, 47]. It has been proposed that augmented activities of the chemoreflex significantly contribute to a lowered activity of the central respiratory drive leading to breathing instability in sleep apnea and also in the pathophysiological impact of sleep apnea mediated by increased sympathetic activities leading to hypertension [3]. Indeed, denervation of the carotid body could normalize the elevated blood pressure induced by intermittent hypoxia in animals [48, 49]. Altered chemosensitivity of the chemoreflex has also been reported in patients with sleep apnea, which significantly contributes to the overactivity of the sympathetic activity mediated by the chemoreflex [50]. Recently, Marcus and colleagues [51] reported that denervation of the carotid body significantly attenuates disordered breathing patterns and renal sympathetic activities in rabbits with cardiac pacing simulating congestive heart failure. The mechanistic cause of the augmented carotid chemoafferent activity is believed to be multifactorial and it could be attributed to the oxidative stress and inflammation induced by intermittent hypoxia [47, 52, 53]. In addition, maladaptive changes in the paracrine-autocrine signaling in the carotid body have been proposed to be responsible for the pathogenic response to intermittent hypoxia [12].

Intermittent hypoxia induces elevated levels of reactive oxygen species (ROS) and nitrogen reactive species leading to oxidative stress, which causes cellular and tissue injuries in organs. The oxidative stress induced by intermittent hypoxia has been proposed as a significant factor contributing to the pathogenesis of sleep apnea [54, 55]. Indeed, superoxide dismutase which is mimetic attenuates the augmented carotid chemoreceptor activity induced by intermittent hypoxia, suggesting an involvement of ROS [44]. It has been proposed that proinflammatory cytokines play a role in the increase in cardiovascular morbidities as a pathophysiological consequence of sleep apnea in patients [56]. Supporting this idea, studies have demonstrated elevated levels of circulating proinflammatory cytokines including IL-6, TNF-*α*, and chemokines including MCP-1 in patients with obstructive sleep apnea [57–59]. In addition, it has been shown that proinflammatory cytokines and hypoxia increase the ROS production, involving the mitochondria and NADPH oxidase [60, 61]. As aforementioned, the elevated proinflammatory cytokines in the arterial blood increase the local expression of proinflammatory cytokines and cytokine receptors in the carotid body [15, 29]. Also, proinflammatory cytokines significantly enhance the intracellular calcium response to hypoxia in the chemosensitive glomus cells [15] and the carotid chemoafferent activity [19, 29]. It has been proposed that expressions of proinflammatory cytokines and cytokine receptors in the carotid body play pathogenic roles in the local inflammation and augmented activities of the carotid body under chronic intermittent hypoxic condition which is a hallmark feature of sleep apnea.

## 6. Intermittent Hypoxia Augments Proinflammatory Cytokine Signaling and Local Inflammation in the Carotid Body

Intermittent hypoxia induces a significant increase in the mRNA and protein expression of proinflammatory cytokines (IL-1*β*, IL-6, and TNF-*α*) and cytokine receptors (IL-1r1, gp130, and TNF-r1) in the rat carotid body [18, 30, 52, 62]. The increased expression of proinflammatory cytokines and receptors is localized to lobules of chemosensitive type-I glomus cells and the proportional amount of the cells expressing cytokines and cytokine receptors is significantly increased by a couple of days of intermittent hypoxia and it remains at an elevated level in hypoxia up to a week [18, 30, 52, 62]. Functional studies show that the effect of exogenous IL-1*β*, IL-6, and TNF*α* at 0.01–1 nM concentration dependently increased the intracellular calcium response to hypoxia by 10–40% in the glomus cell. The calcium response is significantly augmented in cells obtained from rats exposed to intermittent hypoxia [18]. These findings suggest that the increase in local expression of proinflammatory cytokines could increase the local release of cytokines under hypoxic conditions. Also the upregulated expression of the cytokine receptors augments the sensitivity of glomus cells to cytokines under hypoxic conditions. Thus these local changes in the cytokine signaling pathway could play a pathogenic role in the local inflammation and augmented activities of the carotid body induced by intermittent hypoxia.

## 7. Oxidative Stress and Inflammation in the Carotid Body in Intermittent Hypoxia

Reports have shown that intermittent hypoxia induces oxidative stress and inflammation in the carotid body. Hence, there are significant elevated levels of markers of oxidative stress including 8-isoprostane, malondialdehyde, and nitrotyrosine in the serum and the carotid body of rats exposed to intermittent hypoxia equivalent to a severe condition of sleep apnea for days to weeks [52, 63]. In addition, there are significant increases in the macrophage infiltration and the expression of chemokines (MCP-1, CCR2, MIP-1*α*, MIP-1*β*, and ICAM-1) in the carotid body [18, 52]. As mentioned, oxidative stress could lead to cellular injuries and inflammation, which could in turn induce the inflammatory response that could be an important contributing factor in the altered function of the carotid body in intermittent hypoxia. Indeed, the transcriptional upregulation of the chemokines could increase the invasion of immune cells in the carotid body participating in the inflammatory process. Thus, the augmented expression of chemokines induced by intermittent hypoxia plays a role in the inflammation of carotid body, which could also be mediated by oxidative stress induced by intermittent hypoxia.

Mechanistically, NADPH oxidase-dependent ROS generation has been shown to induce oxidative stress in local tissues and increase expressions of proinflammatory cytokines and chemokines via activation of NF*κ*B pathway [64, 65]. Indeed, gene transcripts of NADPH oxidase subunits (gp91^phox^ and p22^phox^) are significantly increased in the carotid body in intermittent hypoxia [18, 63]. Importantly, treatment of anti-inflammatory drugs dexamethasone or ibuprofen significantly attenuates levels of oxidative stress and gp91^phox^ as well as macrophage infiltration in the carotid body of the rat in intermittent hypoxia [18]. Thus, the upregulated proinflammatory cytokine pathway could be initially activated by tissue hypoxia [18, 29]. In turn, the inflammatory response recruits infiltrated macrophages which also contribute to the inflammatory cascade in the carotid body. More importantly, increased levels in the ROS and oxidative stress induced by intermittent hypoxia regulate the gene expression of inflammatory cytokines, chemokines, and adhesion molecules [66]. As a result, these significantly contribute to the upregulated cytokine pathways and local inflammation induced by intermittent hypoxia in the carotid body. Moreover, proinflammatory cytokines, including TNF*α* and IL-1*β*, and HIF-1*α* also cause the activation of NF*κ*b [67, 68]. The interplay of these molecules and signaling pathways could lead to a positive regulatory loop that may further enhance and exaggerate the local inflammatory response in the carotid body. In fact, administration of anti-inflammatory drug dexamethasone or ibuprofen could attenuate the levels of macrophage infiltration and oxidative stress in the carotid body of rats exposed to intermittent hypoxia [18]. Also, ibuprofen prevents the elevated ventilatory response to hypoxia and arterial blood pressure in intermittent hypoxia, although it fails to normalize the augmented carotid chemosensory response to hypoxia [52, 62]. Thus, the activation of proinflammatory cytokine pathway could play mechanistic roles in mediating the local inflammation and in contributing to the altered function of the carotid body in intermittent hypoxia, leading to the pathophysiology and pathophysiological consequences of sleep apnea [12].

Besides, it has recently been shown that blockade of angiotensin II AT_1_ receptors with losartan significantly attenuates the macrophage infiltration, oxidative stress, and inflammation in the carotid body in intermittent hypoxia [69]. There is an upregulation of the expression of a local renin angiotensin system in the carotid body in intermittent hypoxia, which significantly contributes to inflammation and oxidative stress [69]. Marcus et al. [63] also reported that losartan attenuates the expression of gp91^phox^ and superoxide production in the rat carotid body in intermittent hypoxia. Also, activation of gp91^phox^ mediates ROS production in the carotid body via AT_1_ receptor [70, 71]. These findings support a pathogenic role of NADPH oxidase regulated by AT_1_ receptors in the local inflammation of the carotid body. In effect, the upregulation of AT_1_ receptors in the carotid body could increase the NADPH oxidase-mediated ROS production, which exaggerates the inflammation and oxidative stress induced by intermittent hypoxia.

## 8. Perspectives of the Inflammation of the Carotid Body-Clinical Implications in Sleep Apnea

In premature infants, altered functions of the carotid body have been known to be a key drive of the unstable breathing leading to apnea of prematurity. The clinical conditions are commonly associated with inflammation and infection. It has been proposed that the inflammation of carotid body could play a role in the changes in the chemosensitivity of the carotid body, which adversely affects the stability of breathing in the infant [72]. Thus, targeting the inflammation and the altered carotid body function may hint to a novel therapeutic strategy for treating the sleep apnea and its pathophysiological consequence. As shown in experimental studies, anti-inflammatory drug prevents the inflammatory response of the carotid body to intermittent hypoxia [18] and also the elevated ventilatory response to hypoxia and arterial blood pressure in intermittent hypoxia, despite the fact that the drug effect is less significant on the augmented chemoreceptor activity [52, 62]. Nevertheless, these findings suggest that local inflammation of the carotid body plays a role in the altered chemoreflex induced by intermittent hypoxia, although the inflammatory response may not be the sole mechanism responsible for the augmented activity of the chemoreceptor. Given that there are significant elevated levels of the expression of proinflammatory cytokines TNF-*α* and IL-6 and the expressions of endothelin-1 and angiotensin II in the carotid body [18, 30, 52, 62, 69, 73], the augmented carotid chemoreceptor response to hypoxia could be mediated by the paracrine-autocrine signaling molecules involving upregulated cytokine pathways, which contribute to the modulation of the chemoreceptor activity in intermittent hypoxia [12].

In patients, anti-inflammatory medications could be useful to alleviate the inflammation of the upper airway and also to reduce the adenoidal size in children with obstructive sleep apnea [74]. In clinical studies with small groups of adult patients, administration of etanercept, a TNF-*α* antagonist, significantly decreases the apnea-hypopnea index (AHI) and IL-6 levels in the patient [75]. Also sleep apnea is less common in patients with spondyloarthritis who received TNF-inhibitor therapy [76]. In contrast, the AHI is worsened after steroid treatment in the patients for 3 months [77]. Also oral indomethacin administration increases AHI by about 2-fold in healthy patients with mild obstructive sleep apnea, which could be related to reductions in cerebral blood flow and its response to CO_2_ [78]. Thus, clinical studies are limited on the efficacy and effectiveness of the anti-inflammatory drugs in the patient with sleep apnea. The effect of the medication is highly dependent on the complex nature of the etiology and pathophysiological cause of sleep apnea in patients. As compared to the findings in experimental animals, the inflammatory response is more related to the impact of intermittent hypoxia equivalent to a severe level of AHI. Future clinical studies in large patient group with stratification of the inflammatory status and chemosensory function may be useful to address the therapeutic potential of targeting inflammation in the carotid body in sleep apnea.

## 9. Summary and Conclusion

**Figure 1:**
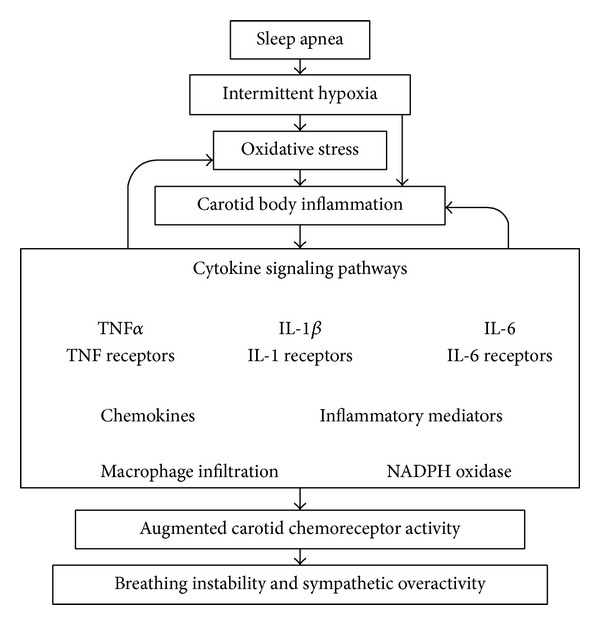
Schematic diagram shows the role of inflammation in the carotid body in the pathogenic cascade of sleep apnea. Arrows are the direction of the pathogenic effects of oxidative stress and inflammation in the carotid body leading to augmented activities of the carotid chemoreceptor and chemoreflex activity, significantly contributing to the pathophysiological consequence impacting on breathing instability and autonomic imbalance.

The expression of proinflammatory cytokines and inflammatory mediators in the carotid body plays a pathogenic role in sleep apnea. As shown in Figure 1, the cytokine signaling pathway is part of the mechanistic cascade involved in the augmented carotid chemoreceptor activity because of the local upregulation of the proinflammatory cytokines, cytokine receptors, and inflammatory mediators in the carotid body. This in effect could significantly contribute to the increased chemosensory activity and the afferent activity of the chemoreflex, causing breathing instability in sleep apnea. In addition, the increased chemoafferent activity could lead to an increase in the sympathetic activity via the chemoreflex. This in turn could significantly contribute to the pathophysiological consequence of the sleep apnea, namely, endothelial dysfunction leading to arterial hypertension and increased risks for cerebrovascular and cardiovascular disease. In experimental animals, studies have shown that anti-inflammatory drugs could significantly attenuate the inflammation of the carotid body and the augmented carotid chemoreceptor activity induced by chronic intermittent hypoxia. In parallel to the conventional treatment of patients with continuous positive airway pressure for sleep apnea, pharmacological intervention has been proposed to alleviate the pathophysiological impact of sleep apnea aiming to reduce the cardiovascular morbidities and mortalities in the patients with sleep apnea. To this end, in addition to the antihypertensive drugs, anti-inflammatory drugs may also be implicative according to the recent advance in the experimental study highlighting a pathogenic role of the local inflammation of the carotid body. Also it is well known that oxidative stress induced by intermittent hypoxia exerts a systemic impact on multiple physiological systems and organs, leading to significant levels of systemic and chronic inflammation in patients with sleep apnea. Thus, it is proposed that the anti-inflammatory drugs could have significant effect not only on targeting the locally upregulated cytokine pathways in the carotid body, but also on the systemic inflammation in other systems and organs as well. Future studies in this direction warrant further investigations and clinical trials have yet to be conducted to confirm the effectiveness of the treatment with anti-inflammatory drugs in patients with sleep apnea.

## Figures and Tables

**Table 1:** A summary of main findings in literatures reporting the expression of cytokines and cytokine receptors in the carotid body of rodents, cats, and humans; also changes in the expression level under hypoxic conditions, namely, sustained hypoxia (SH) or intermittent hypoxia (IH) are shown.

	Rat	Mouse	Cat	Human	SH	IH
IL-1*β*	IHC, PCR [15, 18, 29, 30, 52, 62]			IA [24]	+ [15, 29]	+ [18, 30, 52, 62]
IL-1r1	IHC, PCR, WB [13, 15, 18]			MA, IHC [24, 25]	+ [15]	+ [18]
IL-4				IA [24]		
IL-6	IHC, PCR [15, 18, 29, 30]			MA, IA [24, 25]	+ [15, 29]	+ [18]
IL-6R*α*	WB [14]	MA [25]		MA, IHC [24, 25]		
gp130	IHC, WB [14, 15, 18]			MA [25]		
IL-8				IA [24]		
IL-10				IA [24]		
IL-10R		MA [25]				
TNF-*α*	IHC, PCR, WB [15, 18, 28–30, 52, 62]		IHC, PCR [20]	MA, IHC [24, 25]	+ [15, 29]	+ [18, 30, 52, 62]
TNF-r1	IHC, PCR, WB [15, 18, 28]	MA [25]	IHC, PCR [20]	MA [25]	+ [15]	+ [18]
TNF-r2	IHC, PCR, WB [28]	MA [25]	IHC, PCR [20]	MA [25]		

Techniques involved in the study—IA, immunoassay; IHC, immunohistochemistry; MA, microarray; PCR, polymerase chain reaction; WB, western blot; +, increased expression.
